# Clinical and Liquid Biomarkers of 20-Year Prostate Cancer Risk in Men Aged 45 to 70 Years

**DOI:** 10.1001/jamanetworkopen.2025.56732

**Published:** 2026-02-02

**Authors:** Maximilian Lindholz, Robin Bülow, Ivo G. Schoots, Marc Kölln, Saskia Nolte, Georg L. Baumgärtner, Jean-Francois Chenot, Carsten O. Schmidt, Martin Burchardt, Matthias Nauck, Henry Völzke, Mark O. Wielpütz, Tobias Penzkofer, Norbert Hosten, Charlie A. Hamm

**Affiliations:** 1 Department of Radiology, Charité Universitätsmedizin Berlin, Berlin, Germany; 2 Berlin Institute of Health, Berlin, Germany; 3 Institute of Diagnostic Radiology and Neuroradiology, University Medicine Greifswald, Greifswald, Germany; 4 Department of Radiology and Nuclear Medicine, Erasmus University Medical Center, Rotterdam, the Netherlands; 5 Department of General Practice, Institute for Community Medicine, University Medicine Greifswald, Greifswald, Germany; 6 Institute for Community Medicine, Study of Health in Pomerania–Quality in the Health Sciences, University Medicine Greifswald, Greifswald, Germany; 7 Department of Urology, University Medicine Greifswald, Greifswald, Germany; 8 Institute of Clinical Chemistry and Laboratory Medicine, University Medicine Greifswald, Greifswald, Germany; 9 German Center for Cardiovascular Research, Partner Site Greifswald, Greifswald, Germany; 10 Institute for Community Medicine, Study of Health in Pomerania–Clinical-Epidemiological Research, University Medicine Greifswald, Greifswald, Germany

## Abstract

**Importance:**

Prostate cancer incidence is projected to double by 2040, yet optimal risk stratification approaches for screening remain unclear despite recent international endorsements of organized programs that call for risk-adapted algorithms using readily available biomarkers.

**Objective:**

To identify biomarkers for risk-adapted prostate cancer screening in men without prostate cancer.

**Design, Setting, and Participants:**

This cohort study used data from the Study of Health in Pomerania (SHIP), a prospective population-based research initiative in Germany that randomly invited participants aged 20 to 79 years who underwent comprehensive examinations with structured 20-year follow-up. Data were collected from October 17, 1997, through September 14, 2021, with final analysis completed November 16, 2025.

**Exposures:**

Baseline clinical and liquid biomarkers, including body mass index, waist-to-hip ratio, and glycated hemoglobin, together with prostate cancer–specific biomarkers of serum prostate-specific antigen (PSA) and magnetic resonance imaging–derived prostate volume and PSA density.

**Main Outcomes and Measures:**

The primary outcome was long-term prostate cancer incidence. Cumulative incidence functions for prostate cancer and death were treated as competing risks. Cause-specific Cox models were used to estimate risk and assess the association between baseline biomarkers and long-term incidence.

**Results:**

Among 2651 men included in the study (median [IQR] age, 54.0 [48.0-62.0] years), 1482 (55.9%) had low baseline PSA levels (<1.00 ng/mL), with a cumulative prostate cancer incidence of 0.1% (95% CI, 0.0%-0.4%), 0.6% (95% CI, 0.3%-1.2%), and 3.3% (95% CI, 2.1%-4.8%) at 5, 10, and 20 years, respectively. In 958 men (36.1%) with intermediate PSA levels (1.00-3.00 ng/mL), prostate cancer incidence was 1.4% (95% CI, 0.7%-2.3%), 5.0% (95% CI, 3.6%-6.6%), and 11.8% (95% CI, 9.2%-14.8%) at 5, 10, and 20 years, respectively. In 211 men (8.0%) with high PSA levels (>3.00 ng/mL), prostate cancer incidence was 14.5% (95% CI, 10.1%-19.7%), 28.3% (95% CI, 22.2%-34.8%), and 34.8% (95% CI, 27.5%-42.2%) at 5, 10, and 20 years, respectively. Cumulative prostate cancer incidence differed significantly among the PSA groups (*P* < .001). In univariable and multivariable Cox regression, age (hazard ratio [HR], 1.04; 95% CI, 1.02-1.07), PSA (HR, 1.06; 95% CI, 1.04-1.07), and PSA density (HR, 1.41; 95% CI, 1.30-1.52) remained consistently associated with prostate cancer risk, whereas other variables showed either no association or inconsistent results across models.

**Conclusions and Relevance:**

This cohort study found that a low baseline PSA level was associated with low long-term prostate cancer risk among men aged 45 to 70 years, supporting risk-adapted screening with extended intervals for men with low PSA levels.

## Key Points

**Question:**

What is the association of clinical and liquid biomarkers, including prostate-specific antigen (PSA) and PSA density, with long-term prostate cancer risk in healthy men aged 45 to 70 years?

**Findings:**

In this cohort study of 2651 men without prostate cancer, older age, PSA, and PSA density were associated with an increased hazard of developing prostate cancer. Men with baseline PSA less than 1.00 ng/mL had a 20-year risk of 3.3%.

**Meaning:**

These findings suggest that a low baseline PSA level in midlife is associated with a substantially reduced risk of prostate cancer for up to 20 years, supporting risk-adapted screening strategies that reduce unnecessary procedures.

## Teaser

This cohort study examines the association of clinical and liquid biomarkers, including prostate-specific antigen level and density, with long-term prostate cancer risk in men aged 45 to 70 years.

## Introduction

Prostate cancer is the second most common cancer in men worldwide, with incidence expected to double by 2040.^1^ Early diagnosis substantially enhances treatment outcomes, with 5-year survival rates exceeding 98% in some nations.^2,3^ Prostate-specific antigen (PSA)–based screening has been shown to reduce prostate cancer–specific mortality in men aged 55 to 69 years.^4,5^ However, the adoption of unstructured PSA testing, also named opportunistic PSA screening, is particularly prone to overdiagnosis of insignificant prostate cancer and leads to medical harm, thereby lowering screening benefits.^6,7^ In early diagnosis, magnetic resonance imaging (MRI) after PSA testing has proven to be effective and cost-efficient, reducing overdiagnosis and unnecessary biopsies.^8,9,10,11,12^ However, the effectiveness of this guideline-compliant MRI pathway remains limited, with up to 50% of MRIs showing false-positive findings.^13^ In population-based screening studies, such as the Göteborg-2 (55%), OPT (58%), and PROBASE (69%), this false-positive rate is even higher.^9,14,15,16^ Thus, the Council of the European Union called for identifying feasible biomarkers for accurate stratification to allow for multimodal, risk-adapted prostate cancer screening.^17^

The etiology of prostate cancer remains largely unknown. Established risk factors are nonspecific (eg, age, race and ethnicity, family history), and it is unclear which lifestyle changes, if any, could meaningfully reduce risk.^18^ The association between prostate cancer incidence and factors indicative of metabolic syndromes, eg, abdominal obesity, hyperglycemia, and hypertriglyceridemia, remains controversial. A meta-analysis did not find any significant association, with varying results across geographic regions.^19^ However, increased waist circumference has been linked to higher risk.^19,20^ These conflicting findings underscore the importance of evaluating potential risk factors for prostate cancer in population-based cohorts to assess their clinical relevance and potential utility.

The Study of Health in Pomerania (SHIP) is a prospective population-based research initiative in Germany with long-term follow-up and comprehensive diagnostic testing (eg, whole-body MRI, laboratory assessments, clinical examinations).^21,22^ Using these data, we analyzed prostate cancer incidence over 20 years in men without prostate cancer aged 45 to 70 years,^23^ examining associations with clinical and liquid biomarkers to identify potential markers for risk-adapted prostate cancer screening.

## Methods

### Study Design

This cohort study analyzed prospectively collected data from the ongoing, government-funded, population-based SHIP. The SHIP is an epidemiologic research project in northeast Germany designed to investigate the incidence of various diseases. We used data from 2 independent cohorts: SHIP START (participant enrollment, 1997-2001) and SHIP TREND (participant enrollment 2008-2012). Written informed consent was obtained from all participants per the principles of the Declaration of Helsinki.^21^ Institutional review board approval was granted by the Ethics Committee of the University Medicine Greifswald. Each study proposal was individually reviewed and approved by the study steering committee. This study adhered to the Strengthening the Reporting of Observational Studies in Epidemiology (STROBE) reporting guideline for cohort studies.^24^

**Figure 1.**
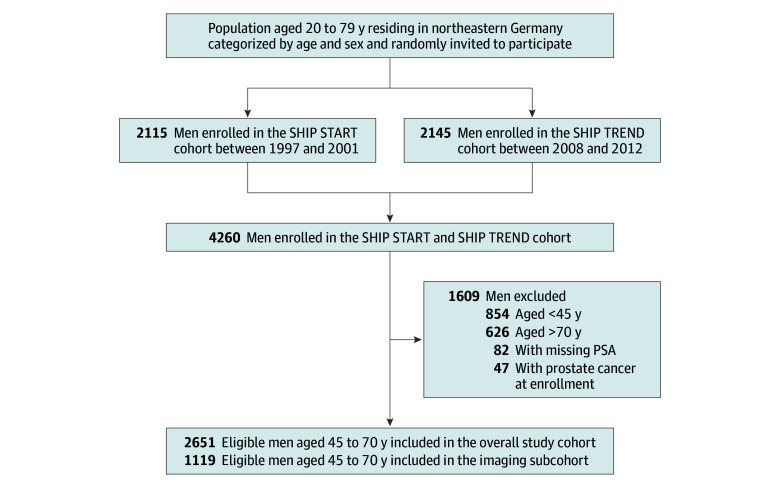
Study Sample PSA indicates prostate-specific antigen.

The comprehensive examination program included whole-body MRI (introduced in 2008), clinical examinations, and laboratory analyses, while continuously integrating data from follow-up visits and health records at 5-year intervals (Figure 1).^21,22^ German citizens aged 20 to 79 years who resided in defined regions of northeastern Germany were randomly invited to participate in the study^21,22^ and stratified by sex and age.

### Study Cohort

This study analyzed longitudinal data of male participants aged 45 to 70 years in the SHIP START and SHIP TREND cohorts. Data on race and ethnicity were not available for this study. This target cohort represents the currently proposed prostate cancer screening population by the European Association of Urology (EAU), including the extended starting age of 45 years instead of 50 years for patients with a family history of prostate cancer.^7,25^ For the SHIP START cohort, MRI data were available from 2008 onward (waves 3, 4, and 5), while no MRI was performed in waves 1 and 2. In the SHIP TREND cohort, all participants were invited to undergo whole-body MRI, and refusal was not considered a protocol violation. Participants with contraindications, such as pacemakers or unclassified implants, did not undergo MRI.

Our analysis used data from the participants’ first visit to capture baseline characteristics for risk stratification. If a participant’s initial visit fell outside the age range of 45 to 70 years, we selected the first subsequent visit within that range as the baseline. For imaging feature analysis, the first visit that included imaging served as the baseline to analyze the effects of imaging features on risk stratification. Outcome data on prostate cancer and mortality were collected from follow-up visits, health insurance claims data, and death registries, with unobserved time considered as censored. Prostate cancer diagnoses were identified using standardized *International Statistical Classification of Diseases, Tenth Revision* codes; however, information on Gleason score is not included in this coding. The first data point in our analysis was on October 17, 1997, and the last was September 14, 2021. Participants with no documented serum PSA or prostate cancer at baseline (diagnosis before or within 4 months after their baseline visit) were excluded from the analysis. Biomarkers obtained during the study were not routinely shared with participants or their health care practitioners, particularly because a subset of markers (including PSA levels in waves 3 and 4 of the SHIP START cohort) was determined retrospectively on prospectively collected samples.

### Study Risk Stratification

Prostate cancer risk was assessed based on clinical and liquid biomarkers, including PSA density calculated from MRI-derived prostate volumes. Clinical biomarkers included age, waist-to-hip ratio, and body mass index (calculated as weight in kilograms divided by height in meters squared). Liquid biomarkers feature glycated hemoglobin, total cholesterol, triglycerides, and high-density lipoprotein cholesterol; red and white blood cell counts, platelet counts, and hemoglobin levels; and serum PSA and PSA density values calculated from MRI-derived prostate volumes. For PSA-based stratification, cutoff groups proposed by the EAU were used (<1.00 ng/mL, 1.00-3.00 ng/mL, and >3.00 ng/mL; to convert to micrograms per liter, multiply by 1).^25^ Additionally, use of the cutoff values introduced by the PROBASE study and proposed by the German prostate cancer guideline, version 8.01 updated in 2025 (<1.50 ng/mL, 1.50-2.99 ng/mL, ≥3.00 ng/mL) were also tested.^16,25^

Whole-body MRI was performed using a 1.5-T scanner. Pelvic imaging was conducted using an axial fat-saturated 2-dimensional proton density turbo spin-echo sequence (repetition time, 3230 milliseconds; echo time, 34 milliseconds; slice thickness, 3 mm; acquisition time, 2.34 minutes).^22,26^ Dedicated prostate MRI, including T2-weighted and diffusion-weighted imaging for early prostate cancer detection, was not acquired. We used a pretrained EfficientNetV2 model for semiautomated prostate volume estimation with a Dice coefficient of 0.94 compared with manual segmentations. The PSA density was calculated by dividing the serum PSA level by the MRI-acquired prostate volume. The PSA density was normalized and mean centered to improve model convergence.

### Study Outcomes

The primary outcome was long-term prostate cancer incidence in men who were aged 45 to 70 years at the time of testing. Prostate cancer was defined as International Society of Urological Pathology grade group 1 or higher. Associations of prostate cancer incidence with clinical and liquid biomarkers were investigated. Furthermore, stratification by age groups was investigated by applying age cohorts defined by the EAU, namely men aged 50 to 59 years or 60 to 70 years.^25^

### Statistical Analysis

The final data analysis was completed on November 16, 2025. Continuous variables were summarized using medians and IQRs. Case analysis was conducted without imputation. Univariable and multivariable cause-specific Cox models were used to analyze the potential biomarkers.^27^ Age and PSA were designated as the primary explanatory variables of prostate cancer, and all multivariable models were adjusted for these factors. Specifically, PSA density and PSA were analyzed with age as a covariate, and conversely, age was analyzed with PSA as a covariate. The Bonferroni-Holm correction was used to adjust for multiple comparisons in regression analyses. The estimative performance of PSA and PSA density for prostate cancer was assessed using time-dependent receiver operating characteristic curves and the area under the curve (AUC) derived from age-adjusted, cause-specific Cox models. Due to the shorter follow-up periods for MRI-derived PSA density, comparisons of the discriminatory ability between PSA and PSA density were conducted in the imaging cohort, using a 12-year estimation period. Cumulative incidence functions accounting for death as a competing risk were estimated to assess the probability of a prostate cancer diagnosis.^28,29^ Gray test was used to compare subdistribution hazards across PSA groups, with post hoc pairwise comparisons conducted between each pair of PSA categories. Statistical analyses were performed using R, version 4.4.1 (R Foundation for Statistical Computing). A 2-sided *P* < .05 was considered statistically significant.

## Results

### Study Cohort

Of the 4260 SHIP participants considered, 2651 men aged 45 to 70 years (median [IQR] age, 54.0 [48.0-62.0] years) were eligible for analysis, including a subcohort of 1119 men undergoing MRI (Figure 1; Table 1). Of the 2651 men, missing baseline data were minimal, ranging from 0 missing data on age and PSA to 68 (2.6%) missing data on hemoglobin. Overall, men had a median body mass index of 28.4 (IQR, 25.9-31.2), a median waist-to-hip ratio of 1.0 (IQR, 0.9-1.0), and a median serum PSA level of 0.88 ng/mL (IQR, 0.57-1.48 ng/mL) (Table 1; eFigure in Supplement 1). In the imaging subcohort, the median prostate volume and PSA density was 35.0 mL (IQR, 29.0-41.9 mL) and 0.03 ng/mL^2^ (IQR, 0.02-0.04 ng/mL^2^), respectively. The median follow-up for the overall study cohort and imaging subcohort was 10.8 years (IQR, 9.0-20.5 years) and 9.7 years (IQR, 6.1-10.8 years), respectively. The cumulative incidence of prostate cancer was 1.8% (95% CI, 1.3%-2.3%), 4.6% (95% CI, 3.8%-5.5%), and 9.1% (95% CI, 7.7%-10.7%) at 5, 10, and 20 years, respectively. For death, the cumulative incidence was 0 (no events) at 5 years, 2.2% (95% CI, 1.6%-2.9%) at 10 years, and 10.2% (95% CI, 8.6%-12.0%) at 20 years.

**Table 1.** Baseline Participant Characteristics

Characteristics	Overall cohort (n = 2651)		Imaging subcohort (n = 1119)	
	Median (IQR)^a^	Missing values, No. (%)	Median (IQR)^a^	Missing values, No. (%)
Age, y	54.0 (48.0-62.0)	0	54.0 (49.0-62.0)	0
Body mass index^b^	28.4 (25.9-31.2)	7 (0.3)	28.1 (25.8-30.8)	0
Waist-to-hip ratio	1.0 (0.9-1.0)	8 (0.3)	1.0 (0.9-1.0)	0
PSA, ng/mL	0.88 (0.57-1.48)	0	0.94 (0.59-1.56)	0
Cholesterol, mmol/L	5.6 (4.9-6.4)	5 (0.2)	5.5 (4.8-6.3)	1 (0.1)
Glycated hemoglobin, %	5.4 (5.1-5.8)	3 (0.1)	5.4 (5.1-5.7)	0
HDL cholesterol, mmol/L	1.2 (1.0-1.5)	8 (0.3)	1.3 (1.1-1.5)	0
Triglycerides, mmol/L	1.7 (1.2-2.6)	4 (0.2)	1.6 (1.1-2.4)	0
Red blood cell count, ×10^6^/µL	4.7 (4.5-5.0)	66 (2.5)	4.8 (4.6-5.0)	29 (2.6)
White blood cell count, ×10^3^/µL	6.0 (5.0-7.2)	2 (0.1)	5.6 (4.8-6.7)	0
Platelet count, ×10^4^/µL	20.8 (17.7-24.2)	66 (2.5)	20.7 (18.0-23.9)	29 (2.6)
Hemoglobin, mmol/L	9.1 (8.6-9.5)	68 (2.6)	9.1 (8.7-9.5)	29 (2.6)
Prostate volume, mL	NA	NA	35.0 (29.0-41.9)	0
PSA density, ng/mL^2^^c^	NA	NA	0.03 (0.02-0.04)	31 (2.8)

Abbreviations: HDL, high-density lipoprotein; NA, not applicable; PSA, prostate-specific antigen.

^a^ SI conversion factors: PSA, multiply by 1 to convert to micrograms per liter; cholesterol and HDL cholesterol, divide by 0.0259 to convert to milligrams per deciliter; glycated hemoglobin, multiply by 0.01 to convert to proportion of total hemoglobin; triglycerides, divide by 0.0113 to convert to milligrams per deciliter; red blood cell count, multiply by 1 to convert to per ×10^12^ per liter; platelet count, multiply by 10 to convert to ×10^9^ per liter; hemoglobin, multiply by 1.61 to convert to grams per deciliter.

^b^ Calculated as weight in kilograms divided by height in meters squared.

^c^ Calculated by dividing PSA levels by prostate volume.

### Univariable Analysis

In the univariable Cox regression analysis, older age (hazard ratio [HR], 1.05; 95% CI, 1.03-1.07; *P* < .001), serum PSA (HR, 1.07; 95% CI, 1.05-1.08; *P* < .001), PSA density (HR, 1.43; 95% CI, 1.33-1.54; *P* < .001), and prostate volume (HR, 1.03; 95% CI, 1.02-1.04; *P* < .001) were each positively associated with prostate cancer risk (Table 2). An inverse, nonsignificant risk for white blood cell count was found (HR, 0.91; 95% CI, 0.83-0.99; *P* = .13), as the corrected *P *value did not cross the predefined confidence threshold. Other clinical and biochemical variables, including body mass index, lipid measures, and hematologic parameters, did not exhibit statistically significant or consistent associations with prostate cancer risk.

**Table 2.** Univariable and Multivariable Competing Risk Analysis of Biomarkers for Increased Long-Term Prostate Cancer Risk

Biomarker	Univariable Cox model^a^		Age- and PSA-adjusted Cox model^a^	
	HR (95% CI)	*P* value^b^	HR (95% CI)	*P* value^b^
Age	1.05 (1.03-1.07)	<.001	1.04 (1.02-1.07)	<.001
Body mass index	0.97 (0.94-1.01)	.24	0.97 (0.93-1.00)	.14
Waist-to-hip ratio	0.15 (0.01-1.96)	.24	0.08 (0.01-1.09)	.14
PSA	1.07 (1.05-1.08)	<.001	1.06 (1.04-1.07)	<.001
Cholesterol	1.03 (0.91-1.17)	.68	1.03 (0.91-1.17)	.72
Glycated hemoglobin	0.89 (0.74-1.06)	.27	0.79 (0.65-0.96)	.06
HDL cholesterol	1.45 (0.98-2.13)	.15	1.41 (0.94-2.10)	.14
Triglycerides	0.89 (0.79-1.00)	.15	0.89 (0.79-1.01)	.14
Red blood cell count	0.85 (0.58-1.25)	.50	1.00 (0.68-1.47)	.99
White blood cell count	0.91 (0.83-0.99)	.13	0.87 (0.79-0.96)	.02
Platelet count	1.00 (0.97-1.03)	.84	1.01 (0.98-1.04)	.72
Hemoglobin	0.81 (0.65-1.02)	.15	0.89 (0.71-1.11)	.38
Prostate volume^c^	1.03 (1.02-1.04)	<.001	1.01 (0.99-1.02)	.37
PSA density^c^	1.43 (1.33-1.54)	<.001	1.41 (1.30-1.52)	<.001

Abbreviations: HDL, high-density lipoprotein; HR, hazard ratio; PSA, prostate-specific antigen.

^a^ Hazard ratios are shown as unadjusted and adjusted for age and PSA. For the variables age and PSA, the adjustments include only each other, and for PSA density, the adjustment includes only age.

^b^ *P* values were adjusted for multiple comparisons using the Holm-Bonferroni method, and all reported *P* values are adjusted.

^c^ Prostate volume was measured on pelvic magnetic resonance imaging, and PSA density was calculated using this volume. These imaging biomarkers were evaluated only in the imaging subcohort (n = 1119), whereas all other variables were assessed in the overall cohort (n = 2651).

### Multivariable Analysis

After adjusting for age (HR, 1.04; 95% CI, 1.02-1.07; *P* < .001) and PSA (HR, 1.06; 95% CI, 1.04-1.07; *P* < .001), both remained associated with higher prostate cancer risk. Prostate-specific antigen density also remained an indicator of prostate cancer risk (HR, 1.41; 95% CI, 1.30-1.52; *P* < .001).

White blood cell count was inversely associated with prostate cancer risk after adjustment (HR, 0.87; 95% CI, 0.79-0.96; *P* = .02). In contrast, prostate volume (HR, 1.01; 95% CI, 0.99-1.02; *P* = .37) and other clinical or biochemical variables, including body mass index, glycated hemoglobin, and lipid measures, showed no clear or significant association with prostate cancer risk.

In addition, we analyzed whether PSA density or PSA is more accurate in assessing prostate cancer risk in the imaging subcohort. The 12-year time-dependent AUC for PSA and age was 0.86 (95% CI, 0.78-0.92), while the AUC for PSA density and age was 0.75 (95% CI, 0.69-0.89). There was no significant difference, but a slightly better performance, of PSA compared with PSA density.

### PSA-Informed Risk Stratification

**Figure 2.**
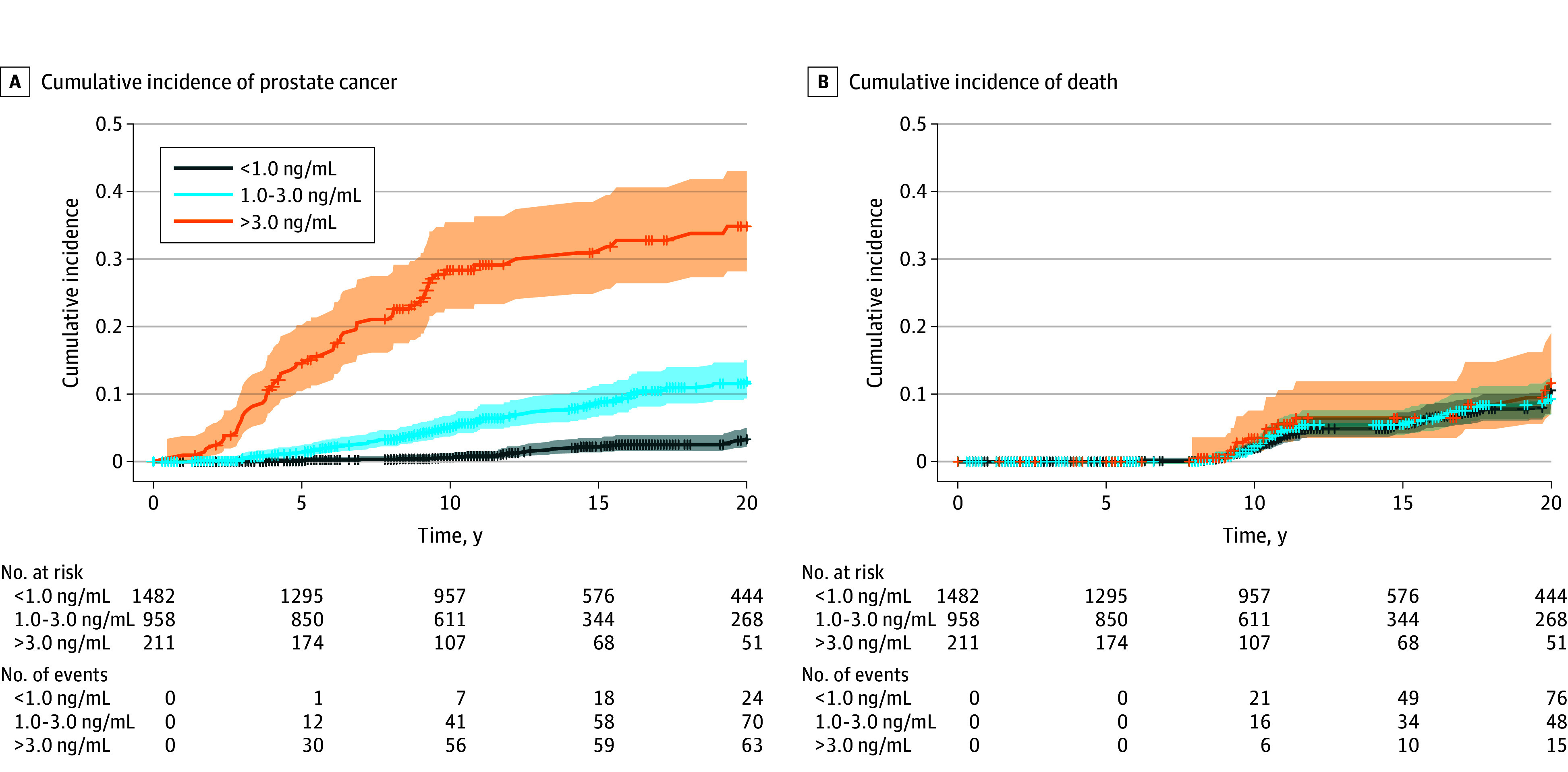
Cumulative Incidence Functions for Prostate Cancer and Mortality in Men Without Prostate Cancer Aged 45 to 70 Years Individual plots represent participants with serum prostate-specific antigen (PSA) levels of <1.00 ng/mL, 1.00 to 3.00 ng/mL, and more than 3.00 ng/mL at baseline (baseline visit), consistent with the risk stratification proposed by the European Association of Urology. Note that the majority of men had a very-low serum PSA (<1.00 ng/mL), which practically excluded prostate cancer over a 20-year period. Shading indicates the 95% CI, and tic marks indicate censoring. To convert PSA values to micrograms per liter, multiply by 1.

Given the association between age and serum PSA, the most commonly used combination of biomarkers for prostate cancer risk stratification, we subsequently examined various established cutoffs and age groups proposed in current screening protocols.^16,25^ A total of 1482 men (55.9%) had serum PSA levels of less than 1.00 ng/mL, while levels of 1.00 to 3.00 ng/mL and greater than 3.00 ng/mL were found in 958 (36.1%) and 211 (8.0%), respectively (Figure 2). Overall, the cumulative prostate cancer incidence differed significantly among PSA groups (*P* < .001 for the overall comparison and all post hoc analyses), whereas mortality did not. Specifically, cumulative prostate cancer incidence at 5 years was 0.1% (95% CI, 0.0%-0.4%), 1.4% (95% CI, 0.7%-2.3%), and 14.5% (95% CI, 10.1%-19.7%) for PSA levels of less than 1.00 ng/mL, 1.00 to 3.00 ng/mL, and greater than 3.00 ng/mL, respectively. At 10 years, the cumulative incidence increased to 0.6% (95% CI, 0.3%-1.2%), 5.0% (95% CI, 3.6%-6.6%), and 28.3% (95% CI, 22.2%-34.8%), respectively. At 20 years, the cumulative incidence further increased to 3.3% (95% CI, 2.1%-4.8%), 11.8% (95% CI, 9.2%-14.8%), and 34.8% (95% CI, 27.5%-42.2%), respectively (eTable 1 in Supplement 1). Using a PSA cutoff of less than 1.50 ng/mL, the cumulative incidence at 5, 10, and 20 years was 0.2% (95% CI, 0.0%-0.5%), 1.1% (95% CI, 0.6%-1.7%), and 4.9% (95% CI, 3.6%-6.4%), respectively (eTable 2 in Supplement 1).

Applying EAU-proposed age groups, men aged 50 to 59 years showed a cumulative prostate cancer incidence at 5 years of 0.2% (95% CI, 0.0%-1.1%), 1.3% (95% CI, 0.4%-3.0%), and 11.1% (95% CI, 4.5%-21.2%) for serum PSA levels of less than 1.00 ng/mL, 1.00 to 3.00 ng/mL, and greater than 3.00 ng/mL, respectively. At 10 years, the cumulative incidence increased to 0.6% (95% CI, 0.2%-1.7%), 5.0% (95% CI, 2.7%-7.9%), and 25.6% (95% CI, 14.4%-38.4%), respectively. At 20 years, the cumulative incidence was 5.2% (95% CI, 2.9%-8.4%), 19.5% (95% CI, 13.4%-26.5%), and 37.4% (95% CI, 22.8%-52.0%), respectively (eTable 3 in Supplement 1). eTables 3 and 4 in Supplement 1 show the cumulative incidences stratified by age (50-59 and 60-70 years) and PSA threshold levels.

## Discussion

The expected global rise in prostate cancer incidence is a pressing health care challenge, necessitating efficient screening strategies. Current opportunistic, repeated, PSA-based prostate cancer screening is susceptible to overdiagnosis of indolent diseases, underscoring the urgent need for improved risk stratification approaches highlighted by the Council of the European Union to mitigate potential medical harm.^30^ This cohort study of a randomly selected prospective population-based cohort found that a single PSA test in men aged 45 to 70 years may effectively identify individuals with a low risk of prostate cancer for up to 20 years. In our sample, 55.9% of men had serum PSA levels less than 1.00 ng/mL, with a low cumulative prostate cancer incidence of 0.1%, 0.6%, and 3.3% over 5, 10, and 20 years, respectively.

Our results align with findings in a Swedish population by Lilja et al,^31^ in which a single PSA measurement before age 50 years could estimate prostate cancer risk up to 3 decades later. In their analysis, 81% of advanced prostate cancer cases (tumor grade T3 or bone metastases) were detected in men with PSA levels above the median (0.63 ng/mL) at age 44 to 50 years. Studies have shown that PSA-based screening effectively lowers the mortality rates associated with prostate cancer, as confirmed by the Göteborg-1, CAP, and ERSPC trials.^5,14,32^ Although the CAP trial indicated that a single PSA screening invitation reduced prostate cancer deaths over 15 years compared with no routine screening, the modest absolute reduction in mortality raises questions regarding the optimal design and setting for such screening strategies. Our findings support and extend current EAU guidelines, which advise against further screening in men aged 60 to 70 years once PSA is less than 1.00 ng/mL.^25^ However, our data provide novel evidence with regard to long-term prostate cancer risk in men aged 50 to 59 years, which may influence screening practices in younger men. Specifically, men aged 50 to 59 years with PSA less than 1.00 ng/mL had very-low long-term prostate cancer incidence, suggesting that extended screening intervals could potentially be safely implemented in this age group as well.

Our findings support transitioning from a traditional rule-in approach to a rule-out strategy. Traditional PSA screening uses a rule-in approach, in which elevated PSA levels prompt additional biopsy evaluations of the prostate. This approach, however, has led to overdiagnosis of indolent disease and numerous unnecessary procedures.^30^ In this context, since most men in our analysis had low PSA levels, many could have been spared from invasive procedures and frequent testing. Moreover, our data specifically support the risk stratification approach currently being evaluated in the PROBASE study, in which men with PSA levels between 1.50 and 2.99 ng/mL receive another PSA test after 2 years, while those with a PSA of 3.00 ng/mL or greater undergo MRI and biopsy.^16^ Although the incidence of prostate cancer after 20 years of follow-up was 10-fold higher in men with PSA greater than 3.00 ng/mL compared with men with PSA less than 1.00 ng/mL, less than half of those with PSA levels greater than 3.00 ng/mL developed prostate cancer during follow-up. This finding underscores the need for improved risk stratification in this group. With 55.9% and 75.0% of men having serum PSA levels of less than 1.00 ng/mL and less than 1.50 ng/mL, respectively, levels that virtually exclude prostate cancer for several years, limited health care resources could be strategically directed to the 8.0% of men with PSA greater than 3.00 ng/mL who are at intermediate and high risk. This targeted approach could substantially improve the efficiency of the diagnostic pathway and potentially reduce unnecessary interventions and health care costs.

While risk-adapted screening potentially enhances oncologic safety and early detection of prostate cancer,^33^ it remains unclear which additional factors physicians might use to stratify patients further. Exploratory analyses showed that lower white blood cell counts were modestly estimative to reduced prostate cancer risk, with consistent inverse trends. Although not statistically significant after correction for multiple testing in univariable analysis, white blood cell counts were associated with risk in multivariable models, indicating a potential independent association that aligns with prior research on white blood cells and prostate cancer.^34^ Moreover, clinical and liquid biomarkers, such as body mass index, lipid levels, and glycemic markers, were either not significant or not consistently associated with prostate cancer risk. As this study did not assess lifestyle interventions, these findings should be interpreted as an absence of detectable associations rather than an absence of causal influence.

Regarding PSA density, several studies have shown that incorporating PSA density into biopsy decision-making increases the diagnostic yield of the MRI pathway.^35,36,37^ However, our findings indicate that it offers no advantage over PSA-informed risk-adapted screening when considering the additional costs of imaging required for PSA density calculation and could directly affect resource allocation in screening programs, suggesting that initial PSA testing alone provides sufficient risk stratification.

### Limitations

Our study has several limitations. While we were unable to distinguish between clinically significant (International Society of Urological Pathology grade group ≥2) and insignificant prostate cancer, the low incidence of prostate cancer in individuals with low (ie, <1.00 ng/mL, <1.50 ng/mL) PSA levels over a follow-up period of up to 20 years supports the utility of this approach in general screening. In addition, given the absence of prostate cancer screening programs in Germany, the prostate cancer cases in our cohort may reflect clinically apparent disease that prompted diagnostic evaluation, representing the type of cases we aimed to detect. Furthermore, the whole-body MRI sequence used is not dedicated to prostate imaging or comparable to the sequences recommended in the Prostate Imaging Reporting and Data System for prostate cancer diagnosis^38^; however, the potential bias introduced regarding prostate volume and the PSA density calculation was consistent across the sample, thereby making results among participants comparable. To maximize interpretability, we restricted our analyses to prespecified Cox models with linear effects and cumulative incidence functions, which provide a transparent and clinically meaningful framework for evaluating biomarker trends in screening. However, we did not explore more complex nonlinear or interaction structures that could be captured by machine learning or ensemble tree-based methods. Finally, available data did not allow us to account for prostate cancer–specific mortality.

## Conclusions

This cohort study of men without prostate cancer aged 45 to 70 years with 20-year follow-up found that a low baseline PSA level was associated with low long-term prostate cancer risk. This finding supports longer screening intervals within population-based, risk-adapted programs. These findings suggest that an initial midlife PSA measurement may help focus resources on individuals at greater risk while reducing unnecessary investigations and overdiagnosis.

## Notes

Supplement 1. **eTable 1.** Cumulative Incidence of Prostate Cancer and Mortality in the Overall Study Sample, Stratified by Baseline Serum Prostate-Specific Antigen Levels as Proposed by the European Association of Urology **eTable 2.** Cumulative Incidence of Prostate Cancer and Mortality in the Overall Study Sample, Stratified by Baseline Serum Prostate-Specific Antigen Levels as Proposed by the PROBASE Study **eTable 3.** Cumulative Incidence of Prostate Cancer and Mortality Stratified by Age Group and Baseline Serum Prostate-Specific Antigen Levels, as Proposed by the European Association of Urology **eTable 4.** Cumulative Incidence of Prostate Cancer and Mortality Stratified by Age Group and Baseline Serum Prostate-Specific Antigen Levels, as Proposed by the PROBASE Study **eFigure.** Distributions of All Variables Shown as Density Plots, Accompanied by a Correlation Matrix Summarizing Pairwise Associations

Supplement 2.
Data Sharing Statement
