# Supplementary material for: Clinical and Liquid Biomarkers of 20-Year Prostate Cancer Risk in Men Aged 45 to 70 Years
Source: JAMA Netw Open. 2026 Feb 2;9(2):e2556732. doi: 10.1001/jamanetworkopen.2025.56732 (PMC12865659; doi:10.1001/jamanetworkopen.2025.56732)
Supplement: Supplement 1. — eTable 1. Cumulative Incidence of Prostate Cancer and Mortality in the Overall Study Sample, Stratified by Baseline Serum Prostate-Specific Antigen Levels as Proposed by the European Association of Urology eTable 2. Cumulative Incidence of Prostate Cancer and Mortality in the Overall Study Sample, Stratified by Baseline Serum Prostate-Specific Antigen Levels as Proposed by the PROBASE Study eTable 3. Cumulative Incidence of Prostate Cancer and Mortality Stratified by Age Group and Baseline Serum Prostate-Specific Antigen Levels, as Proposed by the European Association of Urology eTable 4. Cumulative Incidence of Prostate Cancer and Mortality Stratified by Age Group and Baseline Serum Prostate-Specific Antigen Levels, as Proposed by the PROBASE Study eFigure. Distributions of All Variables Shown as Density Plots, Accompanied by a Correlation Matrix Summarizing Pairwise Associations [file jamanetwopen-e2556732-s001.pdf]

Supplemental Online Content

Lindholz M, Bülow R, Schoots IG, et al. Clinical and liquid biomarkers of 20-year prostate cancer risk in men aged 45 to 70 years. *JAMA Netw Open*. 2026;9(1):e2556732.
doi:10.1001/jamanetworkopen.2025.56732

eTable 1. Cumulative Incidence of Prostate Cancer and Mortality in the Overall Study Sample, Stratified by Baseline Serum Prostate-Specific Antigen Levels as Proposed by the European Association of Urology

eTable 2. Cumulative Incidence of Prostate Cancer and Mortality in the Overall Study Sample, Stratified by Baseline Serum Prostate-Specific Antigen Levels as Proposed by the PROBASE Study

eTable 3. Cumulative Incidence of Prostate Cancer and Mortality Stratified by Age Group and Baseline Serum Prostate-Specific Antigen Levels, as Proposed by the European Association of Urology

eTable 4. Cumulative Incidence of Prostate Cancer and Mortality Stratified by Age Group and Baseline Serum Prostate-Specific Antigen Levels, as Proposed by the PROBASE Study

eFigure. Distributions of All Variables Shown as Density Plots, Accompanied by a Correlation Matrix Summarizing Pairwise Associations

This supplemental material has been provided by the authors to give readers additional information about their work.

© 2026 Lindholz M et al. *JAMA Network Open*.

eTable 1. Cumulative Incidence of Prostate Cancer and Mortality in the Overall Study Sample, Stratified by Baseline Serum Prostate-Specific Antigen Levels as Proposed by the European Association of Urology

Year	Outcome	PSA level (ng/mL)	Cumulative incidence function % (95% CI)	At risk	Event
0	PCa	<1.00	0.0%*	1482	0
5	PCa	<1.00	0.1% (0.0%-0.4%)	1295	1
10	PCa	<1.00	0.6% (0.3%-1.2%)	957	6
20	PCa	<1.00	3.3% (2.1%-4.8%)	444	17
0	PCa	1.00-3.00	0.0%*	958	0
5	PCa	1.00-3.00	1.4% (0.7%-2.3%)	850	12
10	PCa	1.00-3.00	5.0% (3.6%-6.6%)	611	29
20	PCa	1.00-3.00	11.8% (9.2%-14.8%)	268	29
0	PCa	>3.00	0.0%*	211	0
5	PCa	>3.00	14.5% (10.1%-19.7%)	174	30
10	PCa	>3.00	28.3% (22.2%-34.8%)	107	26
20	PCa	>3.00	34.8% (27.5%-42.2%)	51	7
0	Death	<1.00	0.0%*	1482	0
5	Death	<1.00	0.0%*	1295	0
10	Death	<1.00	1.9% (1.2%-2.9%)	957	21
20	Death	<1.00	10.5% (8.3%-13.0%)	444	55
0	Death	1.00-3.00	0.0%*	958	0
5	Death	1.00-3.00	0.0%	850	0

© 2026 Lindholz M et al. *JAMA Network Open*.

Year	Outcome	PSA level (ng/mL)	Cumulative incidence function % (95% CI)	At risk	Event
10	Death	1.00-3.00	2.2% (1.3%-3.5%)	611	16
20	Death	1.00-3.00	9.2% (6.8%-12.1%)	268	32
0	Death	>3.00	0.0%*	211	0
5	Death	>3.00	0.0%*	174	0
10	Death	>3.00	3.4% (1.4%-7.0%)	107	6
20	Death	>3.00	11.6% (6.7%-18.1%)	51	9

Abbreviations: * If no event occurred 95% CI, could not be calculated. PSA= prostate-specific antigen, PCa= prostate cancer, CI= confidence interval; PCa, prostate cancer.

© 2026 Lindholz M et al. *JAMA Network Open*.

eTable 2. Cumulative Incidence of Prostate Cancer and Mortality in the Overall Study Sample, Stratified by Baseline Serum Prostate-Specific Antigen Levels as Proposed by the PROBASE Study

Year	Outcome	PSA level (ng/mL)	Cumulative incidence function % (95% CI)	At risk	Event
0	PCa	<1.50	0.0%*	2000	0
5	PCa	<1.50	0.2% (0.0%-0.5%)	1759	3
10	PCa	<1.50	1.1% (0.6%-1.7%)	1290	14
20	PCa	<1.50	4.9% (3.6%-6.4%)	589	32
0	PCa	1.50-2.99	0.0%*	440	0
5	PCa	1.50-2.99	2.5% (1.3%-4.4%)	386	10
10	PCa	1.50-2.99	8.1% (5.6%-11.1%)	278	21
20	PCa	1.50-2.99	14.7% (10.7%-19.2%)	123	14
0	PCa	≥3.00	0.0%*	211	0
5	PCa	≥3.00	14.5% (10.1%-19.7%)	174	30
10	PCa	≥3.00	28.3% (22.2%-34.8%)	107	26
20	PCa	≥3.00	34.8% (27.5%-42.2%)	51	7
0	Death	<1.50	0.0%*	2000	0
5	Death	<1.50	0.0%*	1759	0
10	Death	<1.50	2.1% (1.5%-3.0%)	1290	32
20	Death	<1.50	10.7% (8.8%-12.9%)	589	75
0	Death	1.50-2.99	0.0%*	440	0
5	Death	1.50-2.99	0.0%*	386	0

© 2026 Lindholz M et al. *JAMA Network Open*.

Year	Outcome	PSA level (ng/mL)	Cumulative incidence function<br>% (95% CI)	At risk	Event
10	Death	1.50-2.99	1.5% (0.6%-3.4%)	278	5
20	Death	1.50-2.99	6.9% (4.1%-10.7%)	123	12
0	Death	≥3.00	0.0%*	211	0
5	Death	≥3.00	0.0%*	174	0
10	Death	≥3.00	3.4% (1.4%-7.0%)	107	6
20	Death	≥3.00	11.6% (6.7%-18.1%)	51	9

Abbreviations: * If no event occurred 95% CI, could not be calculated. PSA= prostate-specific antigen, PCa= prostate cancer, CI= confidence interval; PCa, prostate cancer.

© 2026 Lindholz M et al. *JAMA Network Open*.

eTable 3. Cumulative Incidence of Prostate Cancer and Mortality Stratified by Age Group and Baseline Serum Prostate-Specific Antigen Levels, as Proposed by the European Association of Urology

Year	Outcome	PSA level (ng/mL)	Age group	Cumulative incidence function % (95% CI)	At risk	Event
0	PCa	<1.00	50-59	0.0%*	547	0
5	PCa	<1.00	50-59	0.2% (0.0%-1.1%)	490	1
10	PCa	<1.00	50-59	0.6% (0.2%-1.7%)	357	2
20	PCa	<1.00	50-59	5.2% (2.9%-8.4%)	185	11
0	PCa	1.00-3.00	50-59	0.0%*	341	0
5	PCa	1.00-3.00	50-59	1.3% (0.4%-3.0%)	305	4
10	PCa	1.00-3.00	50-59	5.0% (2.9%-7.9%)	210	11
20	PCa	1.00-3.00	50-59	19.5% (13.4%-26.5%)	78	18
0	PCa	>3.00	50-59	0.0%*	54	0
5	PCa	>3.00	50-59	11.1% (4.5%-21.2%)	47	6
10	PCa	>3.00	50-59	25.6% (14.4%-38.4%)	30	7
20	PCa	>3.00	50-59	37.4% (22.8%-52.0%)	15	4
0	Death	<1.00	50-59	0.0%*	547	0
5	Death	<1.00	50-59	0.0%*	490	0
10	Death	<1.00	50-59	1.7% (0.7%-3.3%)	357	7
20	Death	<1.00	50-59	8.6% (5.7%-12.4%)	185	18
0	Death	1.00-3.00	50-59	0.0%*	341	0
5	Death	1.00-3.00	50-59	0.0%*	305	0

© 2026 Lindholz M et al. *JAMA Network Open*.

Year	Outcome	PSA level (ng/mL)	Age group	Cumulative incidence function % (95% CI)	At risk	Event
10	Death	1.00-3.00	50-59	2.1% (0.8%-4.5%)	210	5
20	Death	1.00-3.00	50-59	6.6% (3.5%-11.1%)	78	7
0	Death	>3.00	50-59	0.0%*	54	0
5	Death	>3.00	50-59	0.0%*	47	0
10	Death	>3.00	50-59	4.1% (0.7%-12.7%)	30	2
20	Death	>3.00	50-59	10.5% (3.1%-23.1%)	15	2
0	PCa	<1.00	60-70	0.0%*	330	0
5	PCa	<1.00	60-70	0.0%*	325	0
10	PCa	<1.00	60-70	1.3% (0.4%-3.2%)	248	4
20	PCa	<1.00	60-70	2.3% (0.9%-4.8%)	147	2
0	PCa	1.00-3.00	60-70	0.0%*	376	0
5	PCa	1.00-3.00	60-70	1.9% (0.8%-3.7%)	366	7
10	PCa	1.00-3.00	60-70	5.5% (3.5%-8.3%)	285	13
20	PCa	1.00-3.00	60-70	9.7% (6.6%-13.4%)	162	10
0	PCa	>3.00	60-70	0.0%*	138	0
5	PCa	>3.00	60-70	17.4% (11.6%-24.2%)	113	24
10	PCa	>3.00	60-70	31.1% (23.4%-39.1%)	70	18
20	PCa	>3.00	60-70	35.4% (26.8%-44.2%)	34	3
0	Death	<1.00	60-70	0.0%*	330	0
5	Death	<1.00	60-70	0.0%*	325	0
10	Death	<1.00	60-70	3.1% (1.5%-5.5%)	248	9

© 2026 Lindholz M et al. *JAMA Network Open*.

Year	Outcome	PSA level (ng/mL)	Age group	Cumulative incidence function % (95% CI)	At risk	Event
20	Death	<1.00	60-70	14.1% (9.7%-19.3%)	147	21
0	Death	1.00-3.00	60-70	0.0%*	376	0
5	Death	1.00-3.00	60-70	0.0%*	366	0
10	Death	1.00-3.00	60-70	3.3% (1.7%-5.6%)	285	11
20	Death	1.00-3.00	60-70	11.1% (7.6%-15.2%)	162	19
0	Death	>3.00	60-70	0.0%*	138	0
5	Death	>3.00	60-70	0.0%*	113	0
10	Death	>3.00	60-70	3.4% (1.1%-8.0%)	70	4
20	Death	>3.00	60-70	12.7% (6.5%-20.9%)	34	7

Abbreviations: * If no event occurred 95% CI, could not be calculated. PSA= prostate-specific antigen, PCa= prostate cancer, CI= confidence interval; PCa, prostate cancer.

© 2026 Lindholz M et al. *JAMA Network Open*.

eTable 4. Cumulative Incidence of Prostate Cancer and Mortality Stratified by Age Group and Baseline Serum Prostate-Specific Antigen Levels, as Proposed by the PROBASE Study

Year	Outcome	PSA level (ng/mL)	Age group	Cumulative incidence function % (95% CI)	At risk	Event
0	PCa	<1.50	50-59	0.0%*	735	0
5	PCa	<1.50	50-59	0.1% (0.0%-0.8%)	661	1
10	PCa	<1.50	50-59	0.8% (0.3%-1.8%)	477	4
20	PCa	<1.50	50-59	7.7% (5.1%-10.9%)	235	21
0	PCa	1.50-2.99	50-59	0.0%*	153	0
5	PCa	1.50-2.99	50-59	2.8% (0.9%-6.6%)	134	4
10	PCa	1.50-2.99	50-59	9.6% (5.4%-15.3%)	90	9
20	PCa	1.50-2.99	50-59	24.1% (14.5%-35.0%)	28	8
0	PCa	≥3.00	50-59	0.0%*	54	0
5	PCa	≥3.00	50-59	11.1% (4.5%-21.2%)	47	6
10	PCa	≥3.00	50-59	25.6% (14.4%-38.4%)	30	7
20	PCa	≥3.00	50-59	37.4% (22.8%-52.0%)	15	4
0	Death	<1.50	50-59	0.0%*	735	0
5	Death	<1.50	50-59	0.0%*	661	0
10	Death	<1.50	50-59	1.8% (0.9%-3.2%)	477	10
20	Death	<1.50	50-59	8.0% (5.5%-11.1%)	235	21
0	Death	1.50-2.99	50-59	0.0%*	153	0
5	Death	1.50-2.99	50-59	0.0%*	134	0

© 2026 Lindholz M et al. *JAMA Network Open*.

Year	Outcome	PSA level (ng/mL)	Age group	Cumulative incidence function % (95% CI)	At risk	Event
10	Death	1.50-2.99	50-59	2.0% (0.4%-6.4%)	90	2
20	Death	1.50-2.99	50-59	7.6% (3.0%-15.1%)	28	4
0	Death	≥3.00	50-59	0.0%*	54	0
5	Death	≥3.00	50-59	0.0%*	47	0
10	Death	≥3.00	50-59	4.1% (0.7%-12.7%)	30	2
20	Death	≥3.00	50-59	10.5% (3.1%-23.1%)	15	2
0	PCa	<1.50	60-70	0.0%*	515	0
5	PCa	<1.50	60-70	0.4% (0.1%-1.3%)	507	2
10	PCa	<1.50	60-70	2.5% (1.4%-4.2%)	390	10
20	PCa	<1.50	60-70	4.4% (2.7%-6.8%)	229	6
0	PCa	1.50-2.99	60-70	0.0%*	153	0
5	PCa	1.50-2.99	60-70	0.0%*	134	0
10	PCa	1.50-2.99	60-70	2.0% (0.4%-6.4%)	90	2
20	PCa	1.50-2.99	60-70	7.6% (3.0%-15.1%)	28	4
0	PCa	≥3.00	60-70	0.0%*	138	0
5	PCa	≥3.00	60-70	17.4% (11.6%-24.2%)	113	24
10	PCa	≥3.00	60-70	31.1% (23.4%-39.1%)	70	18
20	PCa	≥3.00	60-70	35.4% (26.8%-44.2%)	34	3
0	Death	<1.50	60-70	0.0%*	515	0
5	Death	<1.50	60-70	0.0%*	507	0
10	Death	<1.50	60-70	3.7% (2.2%-5.7%)	390	17

© 2026 Lindholz M et al. *JAMA Network Open*.

Year	Outcome	PSA level (ng/mL)	Age group	Cumulative incidence function % (95% CI)	At risk	Event
20	Death	<1.50	60-70	14.2% (10.7%-18.1%)	229	33
0	Death	1.50-2.99	60-70	0.0%*	191	0
5	Death	1.50-2.99	60-70	2.6% (1.0%-5.7%)	184	5
10	Death	1.50-2.99	60-70	6.5% (3.5%-10.7%)	143	7
20	Death	1.50-2.99	60-70	11.4% (6.9%-17.1%)	80	6
0	Death	≥3.00	60-70	0.0%*	138	0
5	Death	≥3.00	60-70	0.0%*	113	0
10	Death	≥3.00	60-70	3.4% (1.1%-8.0%)	70	4
20	Death	≥3.00	60-70	12.7% (6.5%-20.9%)	34	7

Abbreviations: * If no event occurred 95% CI, could not be calculated. PSA= prostate-specific antigen, PCa= prostate cancer, CI= confidence interval; PCa, prostate cancer.

© 2026 Lindholz M et al. *JAMA Network Open*.

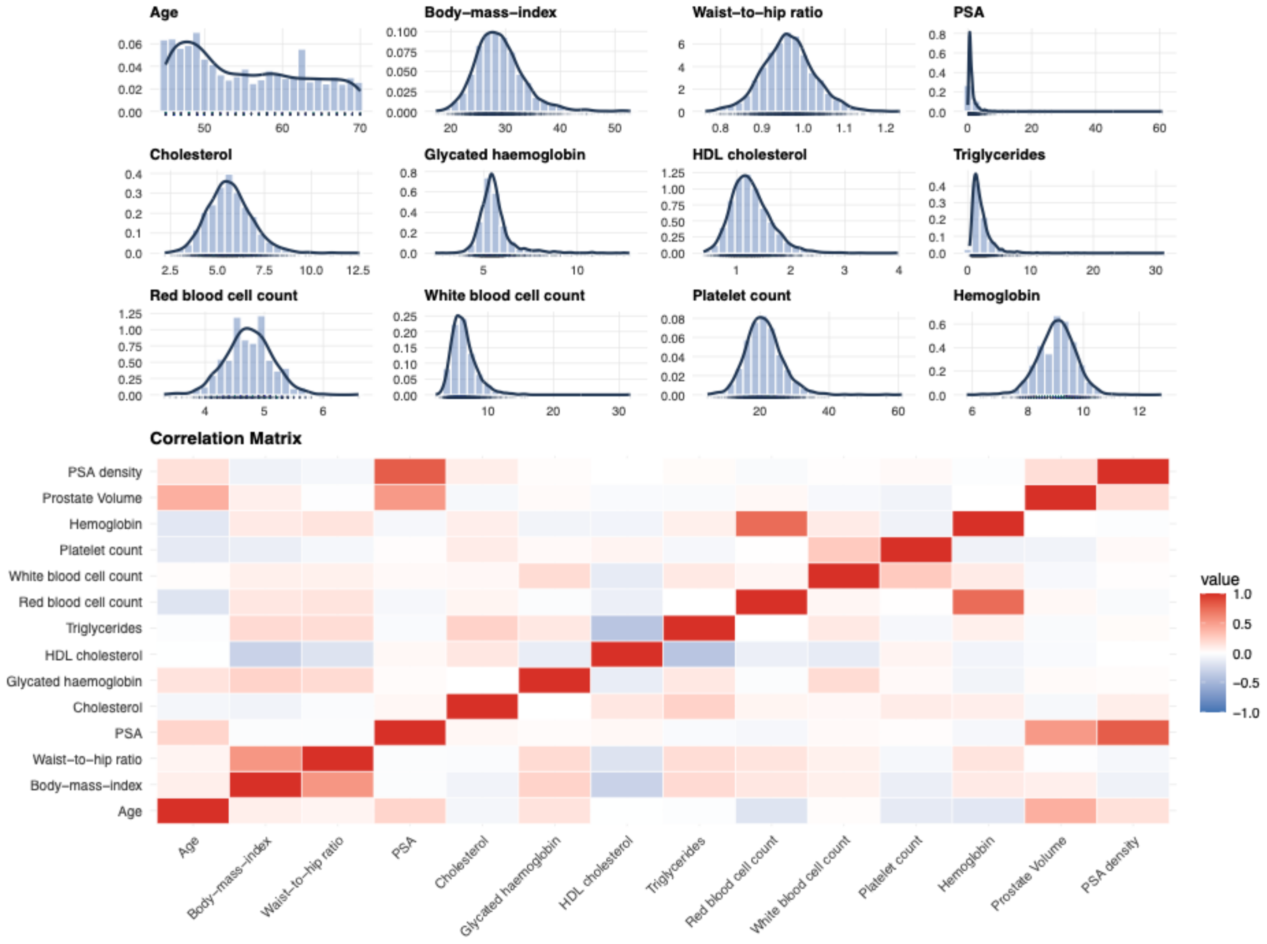

eFigure 1. Distributions of All Variables Shown as Density Plots, Accompanied by a Correlation Matrix Summarizing Pairwise Associations

Age is expressed in years; body-mass-index in kilograms per square meter; waist-to-hip ratio as a unitless ratio; prostate-specific antigen (PSA) and PSA density in nanograms per milliliter (ng/mL) and ng/mL/mL, respectively; serum cholesterol, HDL cholesterol, and triglycerides in millimoles per liter (mmol/L); glycated hemoglobin (HbA1c) in percent; hemoglobin in millimoles per liter (mmol/L); red and white blood cell counts in $\times 10^6$/µL and $\times 10^3$/µL, respectively; platelet count in $\times 10^4$/µL; and prostate volume in milliliters (mL). PSA density was calculated by dividing PSA levels by prostate volume.

© 2026 Lindholz M et al. *JAMA Network Open*.
