# Supplementary material for: Clinical and Liquid Biomarkers of 20-Year Prostate Cancer Risk in Men Aged 45 to 70 Years
Source: JAMA Netw Open. 2026 Feb 2;9(2):e2556732. doi: 10.1001/jamanetworkopen.2025.56732 (PMC12865659; doi:10.1001/jamanetworkopen.2025.56732)
Supplement: Supplement 2. — Data Sharing Statement [file jamanetwopen-e2556732-s002.pdf]

Data Sharing Statement

Data
Data available: No

Additional Information
Explanation for why data not available: The here analyzed data of the study of health in Pomerania (SHIP) are available upon reasonable request from the transfer unit for data and biomaterials at the University Medicine Greifswald, Germany. For any specific inquiries or collaboration requests, please contact the corresponding author.
